# Risk Factors for Dysphagia Following a Cervical Fusion in a Trauma Population

**DOI:** 10.7759/cureus.3489

**Published:** 2018-10-24

**Authors:** Kathryn Cameron, Michael H Lawless, Robert Conway, Gijong Paik, Doris Tong, Teck M Soo, Peter P Lopez

**Affiliations:** 1 Surgery, Ascension Providence Hospital, Michigan State University, College of Human Medicine, Southfield, USA; 2 Neurosurgery, Ascension Providence Hospital, Michigan State University, College of Human Medicine, Southfield, USA; 3 Research, Michigan Spine and Brain Surgeons, Southfield, USA

**Keywords:** dysphagia, cervical arthrodesis, cervical spine injury

## Abstract

Background

Dysphagia following a cervical fusion is a known complication; however, this has not been examined in the trauma population. We sought to identify risk factors that can be optimized in this population.

Methods

We performed a retrospective chart review on consecutive trauma patients who underwent a cervical fusion from 2014 to 2017 at a single institution with multiple surgeons. We included patients more than 18-years-old who were admitted through the emergency department with a diagnosis of acute cervical injury and underwent a cervical fusion during the same admission. We excluded patients who remained intubated postoperatively or underwent a tracheostomy. The primary outcome was dysphagia as evaluated by a bedside swallow test on postoperative day one by the nursing staff. This was followed by a standardized assessment performed by a speech therapist on postoperative day two in some cases. Variables of interest included sex, age, mechanism of injury, surgical approach, cervical levels, and Charlson comorbidity index. Univariate analysis was also utilized.

Results

Sixty patients met the study criteria. Nineteen patients (31.7%) developed dysphagia postoperatively. Mechanical falls were the most common injury mechanism (80%) and most surgical procedures were performed on the subaxial cervical spine (68.3%). Comparing the dysphagia groups, there was no significant difference among the confounding variables. Patients with dysphagia had an increased length of stay (10.6 ± 6.7 vs. 7.4 ± 3.1, *p* = 0.056) and were more likely to have had an anterior vs. posterior cervical fusion (63.2% vs. 34.1%, *p* = 0.056).

Conclusions

We found no statistically significant risk factors leading to postoperative dysphagia. The objective of this pilot is to find the baseline dysphagia rate and the potential modifiable factors in this unique patient population undergoing cervical fusion procedures.

## Introduction

Cervical spine injuries represent a significant burden to the US healthcare system with an estimated annual incidence rate of 64/100,000 [1-2]. Studies have demonstrated an annual incidence of cervical spine fractures requiring surgical management as 3.1/100,000 [3]. Dysphagia is a well-known complication following anterior cervical discectomy and fusion, which is a common approach for subaxial, anterior spinal column pathology [4-6]. Often this dysphagia is a subclinical event; however, it can lead to pulmonary aspiration, representing a significant cause of morbidity and mortality [7-8]. While the incidence of dysphagia has been well documented postoperatively following elective cervical fusion procedures, it has not been examined in the context of cervical fusion procedures for traumatic indications. Given the acuteness of the condition and comorbidities this population might have—such as older age, a larger array of comorbidities, increased frailty score, and other associated traumatic injuries—these patients may differ clinically from the patients undergoing these procedures on an elective basis for degenerative disc disease.

The purpose of this study was to assess what individual variables are associated with dysphagia and aspiration events in this particular patient subset.

## Materials and methods

To elucidate possible risk factors for both dysphagia and aspiration events following cervical fusion procedures for an acute traumatic injury, we performed a retrospective cohort study on the patients of a two-hospital institution who underwent cervical spinal fusion procedures. Our study was approved by the Institutional Review Board. Strengthening the Reporting of Observational Studies in Epidemiology (STROBE) guidelines were followed for study design and manuscript creation [9]. Using appropriate International Classification of Diseases (ICD)-9 and ICD-10 codes, patients who were admitted through the emergency department (ED) with a discharge date January 1, 2014, and February 15, 2017, were identified. Patients were included if they underwent surgical fixation for their admitting diagnosis during the same hospitalization. Preoperative characteristics were obtained from electronic medical records and included: age, gender, calculated Charlson comorbidity index (CCI), mechanism of injury, initial Glasgow coma score (GCS). Length of hospital stay, surgical procedure, surgical approach, the number of cervical spine levels, diagnoses, treatment for dysphagia, and prior cerebrovascular accidents were also observed. Subsequent encounters were also identified for subsequent enteral tube placement.

The primary outcome of our study was the incidence of dysphagia and the secondary outcome was aspiration pneumonia. Included in primary outcome and diagnosis of dysphagia was a further evaluation of tube gastrostomy/jejunostomy placement and mortality. Subgroup analysis was desired to determine if the diagnosis (i.e., dens fracture, central cord syndrome) had an association with dysphagia severity; however, our sample size was too small to adequately power such analyses.

Dysphagia was assessed postoperative day one by nursing staff, per nursing protocols. If the patient failed a bedside swallow exam, in most instances speech pathology was consulted, and a modified barium swallow was performed. Dysphagia was defined as per clinical documentation by nursing staff and/or a speech-language pathologist. Aspiration pneumonia was defined as pulmonary infiltrates on chest radiographs and a secondary sign of infection such as abnormal white blood cell count, fever or a productive cough [10]. Patients who underwent greater than three-level anterior cervical fusion and discectomy were excluded to avoid confounding due to the disproportionately high number of patients who develop postoperative dysphagia in this population [11].

Descriptive analyses were performed using a scatter plot to assess the violations of assumption. Ordinary regression models were used to predict the effect of the various independent risk factors on the development of a postoperative dysphagia. Categorical variables were analyzed using chi-square or Fisher’s exact tests. Continuous variables were analyzed using student’s t-test. p Values less than or equal to 0.05 were considered statistically significant. SPSS Statistics for Windows, version x (SPSS Inc., Chicago, IL, USA) was utilized for statistical analyses.

A total of 557 patient encounters were identified through medical record query. Of these, 70 patients underwent surgical fixation of an acute cervical spine fracture. Sixty patients met study criteria. Two patients were excluded because dysphagia developed more than two days postoperatively, and this was less likely to be directly related with surgery. Five patients who were not extubated prior to the first postoperative day or underwent a tracheostomy were also excluded as these patients inherently could not tolerate oral intake.

## Results

Patient demographics are presented in Table 1. Nineteen patients (31.7%) developed dysphagia. Falls were the most common injury mechanism (80%) and most underwent a subaxial surgery (68.3%) (Table 1). Diagnoses for axial trauma included dens fractures, most often type two, atlantoaxial subluxation, and axis bilateral pars interarticularis fracture. Subaxial injuries included traumatic spondylopathy with central cord syndrome, unstable teardrop fracture, facet fracture, and a combination of various fracture subluxations.

Patient characteristics that were not found to have a significant association with dysphagia included gender, age, and pattern of injury (Table 1). Incidence of dysphagia was higher in patients who underwent anterior vs. posterior cervical fusion (63.2% vs. 34.1%, *p* = 0.056) (Table 1). Patients who had postoperative dysphagia had an increased length of stay (10.6 ± 6.7 vs. 7.4 ± 3.1, *p* = 0.056) (Table 1).

**Table 1:** Comparison between non-dysphagia and dysphagia cohorts: univariate analysis MVA: motor vehicle accident; std: standard deviation

n=70	Dysphagia	No Dysphagia	*p*-value
Gender			
Male	14 (35.9%)	25 (64.1%)	
Female	12 (38.7%)	19 (61.3%)	0.809
Age (mean; std)	65.9 (18.0)	62.1 (17.7)	
Median (range)	68.5 (25-90)	64 (19-89)	0.313
Injury Pattern			
Fall from standing	16 (38.1%)	26 (61.9%)	
Fall from height	3 (23.1%)	10 (76.9%)	
MVA	7 (50%)	7 (50%)	
Assault	0	1 (100%)	0.456
Approach			
Anterior	14 (45.2%)	17 (54.8%)	
Posterior	9 (29%)	22 (71%)	
Circumferential	3 (37.5%)	5 (62.5%)	0.469
Primary vs. Revision			
Primary	25 (38.5%)	40 (61.5%)	
Revision	1 (20%)	4 (80%)	0.644
Number of Levels			
0	0	1 (100%)	
1	6 (28.6%)	15 (71.4%)	
2	5 (35.7%)	9 (64.3%)	
3	6 (46.2%)	7 (53.8%)	
4	3 (42.9%)	4 (57.1%)	
5	4 (40%)	6 (60%)	
6	2 (66.7%)	1 (33.3%)	
9	0	1 (100%)	0.899
Length of Stay in Days (mean; std)	13.35 (±9.51)	7.18 (±3.07)	
(median; range)	10 (3-38)	6.5 (3-15)	0.004
Charlson Comorbidity Index (median; std)	3 (0-10)	2.5 (0-7)	0.663
Mortality			
Yes	3 (100%)	0	
No	23 (34.3%)	44 (65.7%)	0.047

The secondary outcome of aspiration pneumonia is shown in Table 2, which includes similar patient characteristics such as gender, age, and pattern of injury.

**Table 2:** Comparison between aspiration and non-aspiration event cohorts: univariate analysis MVA: motor vehicle accident; std: standard deviation

n=70	Aspiration	No Aspiration	*p*-value
Gender			
Male	4 (10.3%)	35 (89.7%)	
Female	1 (3.2%)	30 (96.8%)	0.374
Age (mean; std)	70.4 (16.8)	62.9 (17.8)	
Median (range)	69 (52-88)	66 (19-90)	0.337
Injury Pattern			
Fall from standing	2 (4.8%)	40 (95.2%)	
Fall from height	1 (7.7%)	12 (92.3%)	
MVA	2 (14.3%)	12 (85.7%)	
Assault	0	1 (100%)	0.491
Approach			
Anterior	1 (3.2%)	30 (96.8%)	
Posterior	3 (9.7%)	28 (90.3%)	
Circumferential	1 (12.5%)	7 (87.5%)	0.512
Primary vs Revision			
Primary	5 (7.7%)	60 (92.3%)	
Revision	0	5 (100%)	0.486
Number of Levels			
0	0	1 (100%)	
1	1 (4.8%)	20 (95.2%)	
2	1 (7.1%)	13 (92.9%)	
3	1 (7.7%)	12 (92.3%)	
4	2 (28.6%)	5 (71.4%)	
5	0	10 (100%)	
6	0	3 (100%)	
9	0	1 (100%)	0.486
Length of Stay in Days (mean; std)	24.8 (11.43)	8.29 (4.84)	
(median; range)	20 (13-38)	7 (3-28)	0.001
Charlson Comorbidity Index (median; std)	5 (3.564)	3 (2.103)	0.201
Mortality			
Yes	2 (66.7%)	1 (33.3%)	
No	3 (4.5%)	64 (95.5%)	0.012

All anterior approach surgeries consisted of single or multilevel anterior cervical discectomy and fusions. Posterior approach surgeries for atlantoaxial injuries consisted of posterior atlantoaxial fusion and occiput to subaxial decompression and fusion. Posterior approach surgeries consisted of multilevel posterior cervical decompression and fusions for subaxial injuries. 

## Discussion

Dysphagia is a well-established complication after anterior cervical discectomy and fusions. Prior studies have found that risk factors for perioperative dysphagia included: the presence of cardiovascular dysfunction, endocrine dysfunction, and multilevel surgeries [11-12]. This is the first study to try to identify the risk factors for dysphagia in a trauma population undergoing anterior, posterior, and combined approaches for cervical spine injuries.

Rates of dysphagia vary greatly, especially in the short-term postoperative period, defined as within one week of surgery [13]. Our rate of 32% was within the reported rates described in prior studies investigating a cervical fusion for non-traumatic indications [6]. We did not evaluate dysphagia over a long-term follow-up, as most incidences of morbid outcome from dysphagia, for example, aspiration pneumonia and hypoxic respiratory coma after failure tend to occur earlier in the peri-operative period [7].

Our results showed a trend toward a correlation between dysphagia and anterior approach (*p* = 0.056). It is well documented that an anterior approach to cervical fusion causes increased rates of dysphagia and this should be expected for traumatic indications as well [5,11]. Evaluating the different potential risk factors, such as the mechanism of injury and comorbidity index did not reveal any significant correlation. Liu et al. reported in a meta-analysis that multiple surgical levels and upper cervical spine, defined as C3-4 interspace, were significant risk factors for dysphagia [11]. Sagi et al. hypothesized that extensive dissection rostral to C4 may cause considerable tissue and pharyngeal trauma and irritation, leading to airway complication [14].

Another explanation may be due to unilateral vocal fold immobility due to recurrent laryngeal nerve paralysis from retraction during anterior exposure [15-16]. While the predominant complaint from vocal fold immobility is dysphonia, dysphagia and aspiration are potential sequelae [16]. This may be particularly true in multilevel surgeries that are associated with longer duration of retraction for operative exposure. We did not find similar associations, which may be in part due to small sample size.

Limitations

Limitations of this study include a relatively small sample size, limiting the power of this study. One potential source of bias is the reporting basis. We decided to utilize standardized nursing documentation for recording the incidence of dysphagia. While arguably the nursing staff should accurately report dysphagia, it is possible that dysphagia was not adequately documented and our incidence may have been underreported. The retrospective nature of this study may also lead to inherently biased findings.

## Conclusions

Overall, our findings lead us to conclude that factors we initially thought would specifically affect the trauma population, such as a potential increased comorbidity, did not affect dysphagia rates. Specific risk factors and areas of potential intervention remain unclear. Further directions would include obtaining a larger patient population with an investigation of other potential risk factors, such as duration of surgery. Prospective interventional studies could also be pursued, in particular, implementation of early speech therapy and rehabilitation of patients exhibiting dysphagia with a cost-benefit analysis.
